# Cell Free Tumoral DNA Versus Paraffin Block Epidermal Growth Factor Receptor Mutation Detection in Patients with Non-Small Cell Lung Cancer 

**DOI:** 10.31557/APJCP.2019.20.12.3591

**Published:** 2019

**Authors:** Hanifeh Mirtavoos-Mahyari, Mohsen Ghadami, Adnan Khosravi, Zahra Esfahani-Monfared, Sharareh Seifi, Elaheh Motevaseli, Mihan Pourabdollah, Mohammadhossein Modarressi

**Affiliations:** 1 *Department of Medical Genetics, Faculty of Medicine, Tehran University of Medical Sciences, *; 2 *Tobacco Prevention and Control Research Center, *; 3 *Chronic Respiratory Diseases Research Center, National Research Institute of Tuberculosis and Lung Diseases (NRITLD), Shahid Beheshti University of Medical Sciences, *; 4 *Department of Molecular Medicine, School of Advanced Technologies in Medicine, Tehran University of Medical Sciences, Tehran. Iran. *

**Keywords:** Carcinoma, non-small-cell lung, DNA, epidermal growth factor receptor, cell free DNA, mutation

## Abstract

**Introduction:**

Increasing knowledge about the molecular profile of tumors has led to personalized treatment for achieving better outcomes in patients with nonsmall cell lung cancer (NSCLC). Currently, finding exact somatic genomic changes of tumor has gained great importance. On the other hand, crescendoing needs to actual tumor tissue at different time points during cancer treatment may produce major discomfort for NSCLC patients. Tumor genomes can be reconstructed by information obtained from circulating cell-free deoxyribonucleic acid (cfDNA) of peripheral blood. cfDNA may be represented as a suitable alternative test for epidermal growth factor receptor (*EGFR*) mutation detection in these patients. This study aimed to assess validity of cfDNA in somatic *EGFR* mutation identification in Iranian NSCLC cases.

**Methods:**

Somatic mutation of *EGFR* gene was studied in both tissue specimens and plasma. Then, mutations were detected by polymerase chain reaction(PCR) and sequencing.

**Results:**

We observed a high concordance (90%) between tissue samples and cfDNA for *EGFR* gene mutation. The sensitivity, accuracy, and positive precision value were 90%, 90% and 100%, respectively. A false negative rate of 10% was also demonstrated in this study.

**Conclusion:**

We established sensitive methods for detecting *EGFR *gene mutation which may be very useful in clinical practice.

## Introduction

Lung cancer has been known as the most leading cause of cancer-related deaths worldwide (Siegel et al., 2013), ranking second and fifth in terms of mortality in Iranian men and women, respectively(Amirkhah et al., 2017). About 85% of lung cancers have non-small cell lung cancer (NSCLC) histopathologic features, while rest of them are small cell lung cancer (SCLC) (Ettinger et al., 2012) NSCLC includes 3 main subtypes of adenocarcinoma, squamous cell carcinoma, and large cell carcinoma. Unfortunately, majority of NSCLC patients have been diagnosed in advanced stages (III or IV) and have poor prognosis (Ettinger et al., 2012). Early cancer diagnosis, appropriate personalized treatment, and serial response evaluation can improve lung cancer outcomes. Recently with the improvement in molecular targeted therapies, the determination of exact clinical phenotype and genotype changes have gained a great importance. Several genetic alterations, including gene amplifications and driver mutations in NSCLC have been studied and implicated for diagnosis, treatment selection, response monitoring, and prediction of prognosis (Ludovini et al., 2011). One of the most known and important mutations in NSCLC, especially adenocarcinoma, is epidermal growth factor receptor (*EGFR*) gene mutations. *EGFR* is a transmembrane protein expressed in lung cells. The most important part of *EGFR* is a kinase domain that binds to specific ligands and transfers signals which promote normal cell proliferation. Somatic mutation of its gene leads to the constant activation of the receptor which will cause uncontrolled cell division and proliferation, angiogenesis, adhesion, invasion, tumor progression, and tumoral cell migration (metastasis) (Sharma et al., 2007). Therefore, the identification of *EGF*R gene mutations has a crucial role to improve lung cancer prognosis; whereas, several specific treatments with tyrosine kinase inhibitors (TKIs) are available with brilliant and dramatic clinical outcomes. The* EGFR* gene has 28 exons, and exons 18 through to 21 code for the tyrosine kinase (TK) domain of the receptor. About 100 *EGFR* gene mutations traditionally have been detected by tissue sample from primary tumor site, but tumoral tissue biopsy is not always feasible especially in patients with poor performance. In addition, sometimes, repeated biopsy may be needed to study changes in molecular portrait of tumor or response monitoring. So, easy, rapid, reliable, and valid tests are very necessary in this regard. 

Liquid biopsy or circulating cell free deoxyribonucleic acid (cfDNA) currently has been proposed in NSCLC as a clinical diagnostic tool, prognostic biomarker, and personalized treatment (Myung et al., 2017). On the other hand, cfDNA may be an alternative to tissue biopsy.

Tumoral DNA is released into blood stream by tumoral cells through tumor shrinkage, necrosis, apoptosis, or metastasis (Schwarzenbach et al., 2011) cfDNA detection is technically challenging, revealing the necessity of cfDNA accuracy and feasibility confirmation.

Most studies, especially those in Asian patients, on *EGFR* mutation have reported high concordance between tumoral tissue samples and cfDNA in blood samples (Veldore et al.,2018). Although several studies have evaluated the validity of cfDNA in *EGFR* mutation identification, its validity is still unknown in Iranian patients. In this study, we aimed to present our data on the validation of cfDNA as a diagnostic approach for screening somatic mutations or hotspot mutations in *EGFR* gene (exons 18, 19, and 21) among NSCLC patients. To best of our knowledge, this is the first study performed on the validation of cfDNA In Iran. 

## Materials and Methods

This retrospective, observational, and single-institute study was carried out on 20 NSCLC histologically confirmed patients at advanced stage IV (Edge and Compton., 2010). It was performed between September and November 2018. These patients were referred to the National Institute of Tuberculosis and Lung Disease (NRITLD), Masih Daneshvari Hospital, a referral hospital in Tehran, Iran. Informed written consent was obtained from each of the patients. Before initiating this study, it was approved by Shahid Beheshti Medical University’s Ethics and Scientific Committees, and it was conducted in compliance with the Helsinki Declaration. Never smoker was used for patient who had smoked less than cigarettes in his/her lifetime (DiFranza et al., 2002) The main end point of this study was concordance between cfDNA and paraffin block of tumoral tissue. Presence or absence of EGFR mutation both in cfDNA and paraffin block of tumoral tissue was defined as concordance.

*Eligibility criteri*a

Patients who aged ≥ 18 years old with histologically confirmed non- squamous NSCLC, at Eastern Cooperative Oncology Group (ECOG) (Oken et al., 1982) performance status (PS) 0-2 , and stage IIIB and IV were enrolled in this study. Other eligibility criteria were receiving ≤2st line chemotherapy, having both tissue and serum samples available, and their organs should have adequate function. 

*Sample collection and DNA extraction*

Formalin fixed paraffin embedded (FFPE) tissues (biopsies or surgically resected specimens), from primary tumors, were used for the mutation analysis at the initial diagnostic procedure for NSCLC. Tumour samples for *EGFR* mutation detection consisted of at least five 10 μm unstained sections mounted on a non-charged microscopic slide containing at least 20% tumour tissue. All primary sites of tissue biopsy were lung or pleura. DNA was extracted and exons 18, 19, and 21 of *EGFR* gene were amplified by nested- polymerase chain reaction (PCR) followed by sanger sequencing. Then, 10 mL of peripheral blood was collected and mixed immediately with 400 microliter of EDTA. The blood samples were transported to lab within 12 hours for further processing. For DNA extraction, the blood samples were centrifuged at 2,500 g for 10 minutes at 4°C. The supernatant was transferred to a new tube and centrifuged at 15,800 ×g for 15 minutes at 4°C. The plasma supernatant was stored at −80°C. The cell free DNA from 1.5 mL plasma was extracted with QIAamp Circulating Nucleic Acid Kit (Cat no: 55114) according to the manufacturer’s instructions.

Detection of EGFR mutations in plasma cfDNA were determined by nested-PCR followed by sequencing with ABI 3,500xl DNA Sequencer. All procedures were performed according to the manufacturer’s instructions. 

## Results

*Patients’ characteristics*

Twenty patients with documented EGFR mutation in paraffin block of tumoral tissue were eligible for this study. Patients’ characteristics are shown in Table 1. Out of 20 patients, 8 were female and 12 were male. Patients’ median age was 60 years old (mean: 59.85±13.44 years old ranging from 38 to 82 years old). The most exon which carried mutation was 19. One patient received Erlotinib as second line systemic chemotherapy after Pemetrexed and Carboplatin, but the rest of the patients were treated with Erlotinib as the first line. The overall rate of objective response to initial *EGFR-TKI* treatment was 95% (n=19), with a median progression-free survival of 11.7 months. Only, one patient (ID number: 94-1343) had a minor single nucleotide polymorphism (SNP) at C.2508 C>T deletion in exon 19.

*Consistency of primary activating EGFR mutation between tissue and plasma*

**Figure 1:**
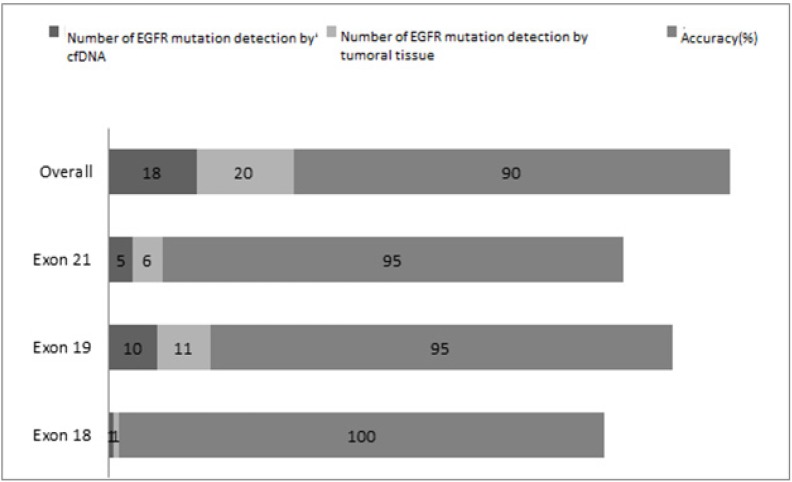
Concordance Analysis in 20 Samples by cfDNA and Tumoral Tissue

The results relating to the consistency of primary active EGFR mutation status with tumor and plasma cfDNA are shown in Figure 1 and Tables 2 and 3. Out of 20 patients who had detectable activating *EGFR* mutation in their tumor tissue, 18 patients exhibited mutation in their cfDNA from plasma. All cfDNA mutation types in these patients were consistent with the primary *EGFR* mutation status on tissue detected before treatment.

**Table 1:** Patients’ Clinical and Demographic Information

	*EGFR* ^a^ exon 18 mutated^b^ patients N (%)	*EGFR* exon 19 mutated ^b^ patients N (%)	*EGFR* exon 21 mutated^b^ patients N(%)	P- value
Gender				
Male	1 (100)	6 (46.1)	5 (100)	0.55
Female	0	7 (53.9)	1	
Total	1	13	6	
Smoking				0.13
Yes	-	2 (13.3)	3 (60)	
No	1 (100)	11 (72.7)	3 (40)	
Total	1	13	6	
Age				
Mean± SD^c^	43	57.53±12.02	66.8±16.51	0.189

^a^ *EGFR*, epidermal growth factor receptor; ^b^, calculated based on both tissue or cfDNA; ^c^SD, standard deviation.

**Table 2:** Performance Metric for cfDNA and Paraffin Block Tumoral Tissue

		Tissue		Sensitivity(%)	Specificity(%)	Accuracy(%)	PPV ^a^(%)
cfDNA^b^		+	-				
*EGFR* ^c^exon 18 mutated patients	+	1	0	100.0	100	100.0	100
	-	0	19				
*EGFR* exon 19 mutated patients	+	12	0	92.3	100	95.2	100
	-	1	8				
*EGFR *exon 21 mutated patients	+	5	0	83.3	100	95.0	95
	-	1	14				
Overall	+	18	0	90.0	-	90.0	100
	-	2	0				

^a^PPV, positive precision value; ^b^cfDNA, cell free DNA; ^c^*EGFR*, epidermal growth factor receptor

**Table 3:** List of True Positive in Both Tissue Samples and cfDNA

ID number	Chromosome	Details	Exon
95-1748	7	C. Substitution of G for Tat nucleotide 2085-P.amino acid Substitution S695R	18
96-3769	7	Deletion of 15 nucleotides at exon 19 (2235-2249) in frame deletion (746-750)	19
96-3896	7	Mutation detected at C.2573 T>G with amino acid change P.L 858 R	21
95-49	7	Deletion of 15 nucleotides at exon 19 (2235-2249) in frame deletion (746-450)	19
9761	7	Mutation detected at C.2573 T>G with amino acid change P.L858 R	21
97-4002	7	Deletion of 15 nucleotides at exon 19 (2235-2249) in frame deletion (746-750)	19
94-1343	7	A single nucleotide polymorphism (SNP) at C.2508 C>T detected	21
96-1774	7	Deletion of 18 nucleotides at exon 19 (2240-2257) in frame deletion (747-753)	19
97-619	7	Deletion of 15 nucleotides at exon 19 (2235-2249) in frame deletion (746-750)	19
96-673	7	12 nucleotides deletion, C.(2240-2251), in-frame deletion (747-751) and insertion of a Serine residue	19
97-733	7	Deletion of 15 nucleotides at exon 19 (2235-2249) in frame deletion (746-750)	19
97-996	7	Deletion of 15 nucleotides at exon 19 (2235-2249) in frame deletion (746-750)	19
97-1001	7	Deletion of 15 nucleotides at exon 19 (2235-2249) in frame deletion (746-750)	19
97-702	7	Deletion of 15 nucleotides at exon 19(2235-2249) in frame deletion (746-450)	19
97-8810	7	Deletion of 15 nucleotides at exon 19 (2235-2249) in frame deletion (746-450)	19
97-211	7	Deletion of 15 nucleotides at exon 19 (2235-2249) in frame deletion (746-450)	19
94-563	7	Mutation detected at C.2573 T>G with amino acid change P.L 858 R	21
613944	7	Mutation detected at C.2573 T>G with amino acid change P.L 858 R	21

**Table 4:** False Negative Samples in cfDNA and Repeated Confirmation in Tissue Biopsy

ID number	Chromosome	Details	Exon
96-896	7	Deletion of 15 nucleotides at exon 19(2235-2249) in frame deletion (746-450)	19
665913	7	Mutation detected at C.2573 T>G with amino acid change P.L 858 R	21

## Discussion

In this study, we validated our cfDNA mutation detection assay on NSCLC Iranian patients. Our study has very high (94.7%) concordance between tumor tissue DNA and cfDNA for *EGFR* mutation detection (Exons 18, 19, and 21 mutations). To best of our knowledge, the current study provided the first data in Iran assessing the feasibility of detecting *EGFR* mutation in the blood sample. 

The concordance rate of *EGFR* mutation between cfDNA and tumor tissues depends heavily on detection methods, ranging from 31% to 100% (Brevet et al., 2011; Zhang et al.,2018; Kimura et al.,1992; Wu et al., 2017). Similar to our result, Kimura et al., (2007) studied 42 patients with NSCLC who were treated with TKIs, and found a concordance rate of 92.9% with a sensitivity of 85.7% using PCR for cfDNA detection. In another large study on 652 patients with NSCLC who were treated by TKIs, the sensitivity and specifity were 65.7% and of 99.8%, respectively (Douillard et al., 2014)Two large meta-analyses comprising 20 and 25 studies, respectively (Luo et al., 2014; Kimura et al.,1992). compared the performance of cfDNA and tissue for *EGFR* mutation detection and demonstrated pooled sensitivity of 67% for cfDNA and 61% for tissue . In addition, in these studies, cfDNA specificity was 93% and 90%, respectively. Several factors can contributed to this wide variation among different studies, including differences in sample source (e.g., plasma vs serum), different methods in DNA extraction and mutation detection, and heterogeneity of the patient populations.

Every year, 1.8 new cancer patients and 1.6 million cancer related-deaths happen following lung cancer diagnosis (Torre et al., 2015) Although the incidence of lung cancer is still lower in Iran than other western countries, lung cancer-induced mortality ranks second and fifth in Iranian men and women, respectively. Most of newly diagnosed NSCLC are locally advanced (inoperable stage IIIB and IIIC) or metastatic (stage IV) at the time of diagnosis (Khosravi et al., 2017). At present time, NSCLC treatment has been personalized according to the patient’s PS, disease stage, tumor histology, and molecular profile. Several genetic alterations have been reported in NSCLC, but only three of them, namely-*EGFR* mutations, anaplastic lymphoma kinase (ALK)-rearrangements, and c-ros oncogene 1 (ROS1) have been approved for systemic therapy (Duréndez-Sáez et al., 2017). One of the most important mutations, especially in adenocarcinoma, is *EGFR* mutations. The prevalence of* EGFR* mutations ranges from 27% to 60% in East Asians, from 8% to 13% in Europeans, and from 12% to 16% in African and white Americans. In East Asian region, higher mutation rates were reported (47% - 64% among different East Asian countries) (Basi et al., 2017). EGFR gene mutations can lead to amplification and overexpression of EGFR protein as well as other carcinogenic mechanisms of EGFR tyrosine kinase activity disorders. Block of such receptors in NSCLC can inhibit tumoral proliferation or disease progression. Therefore, TKIs are widely used for NSCLC patients who are in stage IIIB–IV and with EGFR gene mutations. Hence, EGFR testing in primary tumor tissue has become a gold standard in clinical practice (Rosell et al.,2012; Mayo-de-las-Casas et al., 2017). The genetic alterations are tested via different techniques, including PCR, next-generation sequencing (NGS), FISH, and IHC in tissue blocks (Jamal-Hanjani et al., 2017). However, in 5%–20% of patients, tumoral tissue biopsy is not always feasible due to insufficient samples, invasive procedure, high cost, or patient’s poor performance (Shiau et al.,2014). Thus, using cfDNA to find EGFR mutations for treatment decision with TKIs has been suggested in clinical decision-making, monitoring of response to treatment, or detection of drug-resistant mutations. On the other hand, the use of cfDNA as a minimally invasive assay for gene mutation study is increasingly appreciated.

cfDNA is referred to short double-stranded DNA fragments in blood or serum (Singh et al., 2017). Presence of cfDNA in the plasma was first described by Mandel and Metais (Mandel,1948) Further studies have shown greater levels of plasma cfDNA than normal controls (Komatsubara and Sacher, 2017). The exact mechanism involved in the release of cfDNA into the blood has not been well understood; nonetheless, multiple mechanisms have been suggested, including extracellular vesicle secretion, tumor cell apoptosis, and necrosis. cfDNA is about ~170 bp and rapidly disappears after surgery or chemotherapy (Hao et al., 2017). The U.S. Food and Drug Administration (FDA) agreed to the EGFR Mutation Test v2, a blood-based companion diagnostic for Erlotinib (Audibert et al., 2016). Similar to tissue samples, cfDNA can be detected by PCR (Allele-Specific PCR, Scorpion Amplified Refractory Mutation System PCR, and Droplet Digital PCR) or NGS. With NGS technology in addition to specific hotspot mutations, novel mutations may be identified (Veldore et al.,2018). Currently, a novel peptide nucleic acid (PNA)-mediated 5’ nuclease PCR (TaqMan) assay has been improved with 78% sensitivity and 100% specificity]. (Duréndez-Sáez et al., 2017; Zhang et al., 2016). *EGFR* mutations in cfDNA isolated from baseline blood samples from patients can be assessed by this method. We chose PCR method because it is a simple and cost-effective technology. In real world, there is no consensus regarding which test is better than the other for *EGFR* mutation detection. However, based on the most important studies such as EURTAC, IPASS, and LUX-Lung, the allele-specific PCR-based assay is an efficient and sensitive test for *EGFR* mutations identification (Rosell et al., 2012; Mok et al., 2009; Sequist et al., 2013).Yung et al., (2009) demonstrated that digital PCR analysis had a high sensitivity of 91.7% for detection of EGFR mutation in plasma samples.

Some studies have expressed other implications for cfDNA. For example, higher cfDNA levels are negative indicator of poor outcome in NSCLC, while declining cfDNA after chemotherapy may be a marker indicating better outcome (Wei et al., 2018). Additionally, quantitative analysis of cfDNA is being used in the prediction of early relapse after treatment (Duréndez-Sáez et al., 2017). *T790M EGFR* mutation is a kind of driver mutation of *EGFR* gene which take places in 50% to 60% of progressed patients (Gainor and Shaw, 2013), resulting in resistance to TKIs treatment in patients who harbor this mutation. To date, the only test that has been approved by the FDA for T790M EGFR mutation detection is the cobas® *EGFR *Mutation test developed by Roche. cfDNA can be used successfully in the detection of *T790M EGFR* mutation based on several studies (Ho et al., 2019). 

In addition, other studies on patients who were treated with immunotherapy agents revealed several benefits for using cfDNA in response monitoring (Goldberg and Patel., 2018).

In conclusion, we have established sensitive methods for detecting *EGFR* gene mutation which may be very useful in targeted therapy planning in NSCLC patients.,.
